# The impact of retractor SPONGE-assisted laparoscopic surgery on duration of hospital stay and postoperative complications in patients with colorectal cancer (SPONGE trial): study protocol for a randomized controlled trial

**DOI:** 10.1186/s13063-016-1256-x

**Published:** 2016-03-10

**Authors:** Alice M. Couwenberg, Maarten J. P. Burbach, Anke B. Smits, Marco Van Vulpen, Wilhemina M. U. Van Grevenstein, Peter G. Noordzij, Helena M. Verkooijen

**Affiliations:** Department of Radiotherapy, University Medical Center Utrecht, Heidelberglaan 100, Utrecht, 3508 GA The Netherlands; Department of Surgery, St. Antonius Hospital, Koekoekslaan 1, Nieuwegein, 3430 EM The Netherlands; Department of Surgery, University Medical Center Utrecht, Heidelberglaan 100, Utrecht, 3508 GA The Netherlands; Department of Anesthesiology, St. Antonius Hospital, Koekoekslaan 1, Nieuwegein, 3430 EM The Netherlands; Imaging Division, University Medical Center Utrecht, Heidelberglaan 100, Utrecht, 3508 GA The Netherlands

**Keywords:** Colorectal cancer, gastrointestinal surgery, laparoscopic surgery

## Abstract

**Background:**

To achieve an adequate visual working field during laparoscopic colorectal surgery without disturbance of the small intestine, patients are positioned in the Trendelenburg position. This position results in hemodynamic changes that may increase the risk of cardiopulmonary complications and prolonged hospital stay. Recently, an intraoperative retractor sponge was introduced as an alternative to the Trendelenburg position during laparoscopic surgery.

The objective of this trial is to study the impact of the use of an intraoperative retractor sponge on the duration of the hospital stay and risk of perioperative complications in patients undergoing laparoscopic surgery for colorectal cancer.

**Methods/design:**

The SPONGE trial is a monocenter study and follows the cohort multiple randomized controlled trial (cmRCT) design. It will be conducted within a multicenter prospective observational cohort of colorectal cancer patients of all stages, for whom longitudinal clinical data and patient-reported outcomes are collected. Patients within the cohort, who will undergo laparoscopic surgery for distal colon or rectal cancer, are eligible for inclusion and form a subcohort. From this subcohort, a 1:1 random sample will be offered to undergo surgery with the use of the retractor sponge. Patients from the subcohort who are not selected will undergo standard treatment, that is, surgery in the Trendelenburg position. The primary endpoint is the duration of the postoperative hospital stay. Secondary outcomes are duration of surgery; intraoperative blood loss and fluid balance; and postoperative body temperature, oxygenation and complications. Both arms require 94 patients.

**Discussion:**

This study is the first randomized controlled trial to evaluate the effect of sponge-assisted laparoscopic colorectal surgery in comparison with standard Trendelenburg position on hospital stay and peri- and postoperative complications. Results of this study will also be relevant for other surgical procedures in the pelvic region. The present study is the second randomized controlled trial according to the cmRCT design, which is embedded within our colorectal cancer cohort.

**Trial registration number:**

ClinicalTrials.gov NCT02574013. Registered 27 September 2015.

## Background

Colorectal cancer is the fourth most common cause of cancer worldwide with the highest incidence in developed countries [1]. Surgery is still the cornerstone of curative colorectal cancer treatment. In the Netherlands, more than half of the colorectal resections is performed laparoscopically [2]. In laparoscopic colorectal surgery, a clear view on the operating field without disturbance of the small intestine in the pelvic region is essential. To achieve this, patients are positioned in a head down (Trendelenburg) position, having gravity retract the intestines away from the pelvic region to obtain a clear working field [3]. The Trendelenburg position is associated with hemodynamic instability due to the increased intrathoracic and intracranial pressure [4–6]. This may complicate anesthetic control and could potentially cause postoperative airway obstruction due to laryngeal edema, and a decrease in postoperative lung function [7–9]. Nevertheless, the effects of the Trendelenburg position on postoperative outcomes, for example, cardiac and pulmonary complications and hospital stay, are still unclear because alternatives for the Trendelenburg position have not been available so far.

Recently, the Endoractor™ (CE Kawamoto Corporation, Osaka, Japan) retractor sponge has been introduced to keep the small intestine aside and create a proper view during laparoscopic pelvic surgery while the patient is in a horizontal position. The initial Dutch experience with the retractor sponge comes from a nonrandomized matched pilot study in which 45 patients underwent sponge-assisted operations and 45 control patients were operated on while in the Trendelenburg position [10]. Patients who underwent sponge-assisted surgery had a shorter hospital stay compared to those in the Trendelenburg position (5.4 and 7 days, respectively, *P* = 0.041). Fewer cardiac complications were observed in the sponge-assisted surgery group compared to the Trendelenburg group, although this difference was not significant (one patient with heart failure versus three patients with heart failure and one patient with myocardial infarction, respectively). These results seem promising; however, confirmation with larger numbers in a randomized setting is required.

Herein, we present a trial designed to evaluate the effect of sponge-assisted laparoscopic colorectal surgery on the duration of hospital stay in comparison with standard care, that is, surgery in the Trendelenburg position. In addition, we will assess the impact of sponge-assisted surgery on postoperative complications, perioperative blood loss and fluid balance, operation time, and postoperative oxygenation and body temperature.

## Methods/design

### Study design

The SPONGE trial is a monocenter randomized controlled trial conducted within a multicenter observational prospective cohort of colorectal cancer patients. This project is launched as the prospective initiative colorectal cancer cohort (Dutch: “Prospectief Landelijk ColoRectaal carcinoom Cohort” (PLCRC)) and follows the protocol of the “ProspectIve data CollectioN Initiative on Colorectal cancer (PICNIC)” (ClinicalTrials.gov: NCT02070146). This cohort was initiated in 2012 at the University Medical Center Utrecht (the Netherlands) and includes colorectal cancer patients of all stages. The cohort follows the “cohort multiple Randomized Controlled Trial” (cmRCT) design, which aims to facilitate multiple pragmatic randomized controlled trials simultaneously [11].

### Cohort informed consent procedure

Patients are enrolled in the cohort after a diagnosis of colorectal cancer. Informed consent for the longitudinal collection of clinical data is required for inclusion. Additionally, patients may provide optional consents to fill-out questionnaires on patient-reported outcomes and for the collection of biomaterials (tumor tissue and blood sample). Upon enrollment, patients are also asked to provide “broad consent for randomization,” according to the staged-informed consent procedure developed for cmRCT [12]. This means that patients may be randomly selected for experimental interventions in the future if eligible for this intervention. These patients will then be offered this experimental intervention, which they may accept or refuse. In case of refusal, patients receive standard care. Only patients who accept the offer will sign an additional informed consent. Patients who are eligible for the experimental intervention but who are not randomly selected will undergo standard care and will not be informed about the experimental intervention. They will serve as controls and their data are compared to the randomly selected patients who were offered the intervention.

### Patient recruitment

For the present study, patients are enrolled in the cohort at the Department of Surgery of the St. Antonius Hospital, Nieuwegein, The Netherlands. Eligible patients meet the following inclusion criteria: 1) histologically confirmed distal colon (sigmoid) or rectal cancer, 2) planned for elective laparoscopic colorectal surgery, 3) performance status WHO 0 to 2 [13], and 4) broad consent for randomization (Table 1). Patients with an indication for open colorectal surgery or emergency colorectal surgery and patients with an inadequate understanding of the Dutch language in speech and/or writing are excluded.

**Table 1:** Inclusion and exclusion criteria for the SPONGE trial

Inclusion criteria	Exclusion criteria
Participant in the PLCRC project	Indication for open colorectal surgery
Informed consent obtained for being offered experimental interventions within the PLCRC project	Emergency colorectal surgery
Distal colon (sigmoid) or rectum carcinoma	Inadequate understanding of the Dutch language in speech and/or writing
Elective laparoscopic surgery	
Performance status WHO 0 to 2	

PLCRC, prospective cohort of colorectal cancer patients; WHO, World Health Organization performance status

### Random selection

**Fig. 1:**
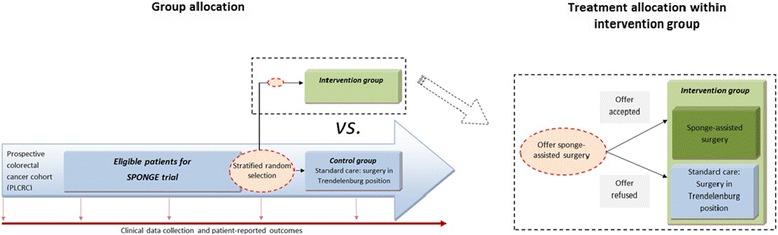
Overview study design and treatment allocation. PLCRC, prospective cohort of colorectal cancer patients; SPONGE trial, randomized controlled trial for clinical evaluation of an intraoperative retractor SPONGE in laparoscopic colorectal surgery

Participants who meet the inclusion criteria of the SPONGE trial form a subcohort of eligible patients (Fig. 1). From this subcohort, patients are randomly selected on a 1:1 basis. Randomly selected patients are offered sponge-assisted surgery. Stratified randomization is performed to take into account participation in other ongoing trials within the cohort, of which the (secondary) endpoints may interfere with endpoints of the SPONGE trial. At this moment, patients are stratified according to whether they received boost radiotherapy in the context of the RECTAL BOOST study, which is running as a cmRCT within the cohort (ClinicalTrials.gov: NCT01951521) [14]. In this trial, a randomly selected group of patients with locally advanced rectal cancer is offered an additional radiation boost prior to standard neoadjuvant chemoradiation. Stratified randomization, using dedicated software, takes place at the Imaging Division University Medical Center Utrecht.

### Study specific informed consent procedure

Patients randomly selected to be offered the sponge-assisted surgery form the intervention group. If these patients accept the offer, additional informed consent is signed to undergo sponge-assisted surgery. Patients who are eligible but who are not randomly selected form the control group and undergo standard treatment, that is, surgery in Trendelenburg position. According to the cmRCT design, patients in the control group will not be informed about the sponge-assisted surgery intervention and will not sign additional informed consent.

### Blinding

This trial is not blinded. The cmRCT design does not enable blinding of participants in the intervention arm. Randomly selected patients will be informed about the sponge intervention and must accept or refuse the sponge-assisted surgery. Patients in the control group are blinded to the fact that they serve as controls in the SPONGE trial. Investigators and physicians cannot be blinded to sponge use because operation reports require indication of the used devices for safety reasons.

### Intervention

The intervention group consists of all patients that have been randomly selected to receive an offer for sponge-assisted surgery. The sponge will be used only in patients who have accepted the intervention. The sponge, the Endoractor™, is a sterile device made of compressed cellulose material, which makes it suitable to fit through a 12-mm port. When inserted into the abdominal cavity, saline increases the sponge size approximately nine times and retracts organs by displacement, weight, and friction. First, the patient is positioned in the Trendelenburg to allow sponge positioning. After placement, the patient is repositioned into a horizontal position. After completion of the surgery, the sponge can be removed easily through a 12 mm port incision.

No side effects or complications related to sponge use were reported in a previous study [15] or in the pilot study. The sponge was tested for potential adverse effects such as cytotoxicity and intracutaneous reactivity [15]. No tissue trauma or organ damage due to the sponge has been reported so far. The retractor sponge is X-ray detectable in case it was left inside the abdomen. The center in which the study is performed handles a strict surgical safety checklist, which includes noting sponge use, making the chance highly negligible that the sponge will not be removed from the abdomen. Presently, the SPONGE trial is being conducted with an experienced colorectal surgeon who has been working with the retractor sponge since 2013. In the near future, other colorectal surgeons will participate. Before participating in the study, surgeons will have completed a learning curve of 10 sponge-assisted surgeries.

### Standard treatment

The control group consists of eligible patients from the subcohort who were not randomly selected to receive an offer of the sponge intervention. During surgery, the control patients are positioned in the Trendelenburg position, which is standard care during laparoscopic colorectal surgery. The surgeon will decide the angle of the Trendelenburg without a pre-specified angle range. Aesthetical, surgical, and pre- and post-supportive care other than the positioning of the patient are not prescribed by this study protocol and are carried out according to the standards used in the hospital.

### Primary outcome

The primary endpoint of this study is postoperative hospital stay, defined as days from surgery until discharge. Discharge needs to be approved by a checklist confirmed by the attending physician. The checklist includes the following items: absence of fever (temperature below 38.5 °C); adequate pain management (VAS score below 4); absence of leukocytosis; passing stool (anally or via stoma); resumption of normal food intake (no nausea or vomiting); absence of an active, unstable and untreated cardiac, pulmonary or surgical complication; and presence of a supportive person at home at the time of discharge [16, 17].

### Secondary outcomes

Secondary endpoints are duration of surgery (minutes), blood-loss (mL), fluid balance (L), body temperature on arrival at the recovery room, oxygen therapy at discharge of the recovery room and postoperative complications (until 30 days after primary surgery).

Pulmonary complications are defined as respiratory failure, requiring mechanical ventilation; atelectasis, pleural effusion, or pneumothorax, as diagnosed on chest radiographs; or pneumonia, based on clinical, laboratory, and imaging findings combined with initiation of antibiotic therapy. Cardiac complications are reported as symptoms of myocardial ischemia and cardiac events, including acute myocardial infarction, congestive cardiac failure, new-onset or rapid atrial fibrillation, major arrhythmia, and cardiac arrest. Surgical complications are reported as symptoms of surgical site infection, anastomotic leakage, postoperative bleeding, or ileal and early stoma-related complications (that is, ischemia, stoma retraction, or small bowel obstruction). All endpoints will be captured from patients’ medical file, operation, and anesthesia records.

### Data collection

Clinical data are being captured from the patients’ electronic medical files and will be collected in an electronic database encompassing data of all cohort participants. In this database, variables are registered under a unique study number provided to each cohort participant. For patients in the SPONGE trial, additional clinical information, that is, duration of surgery, blood loss, fluid balance, postoperative body temperature, and oxygen therapy, will be collected from electronic medical files. Only the research office of the Imaging Division, University Medical Center Utrecht, possesses the key to link study numbers back to patient identifiers. At baseline, data will be collected on demographics, body mass index, co-morbidities, tumor characteristics, neoadjuvant therapy, ASA classification, type of anesthesia, and surgical procedure.

### Sample size

In the previously mentioned pilot study, the hospital stay in the in the Trendelenburg group was 7.0 days compared to 5.4 days in the sponge-assisted surgery group, with a standard deviation of 2.04 [10]. To calculate a sample size, we used a two-sided test with a type I error of 5 % and power of 80 %. We assumed a difference in duration of hospital stay of 1 day to be clinically relevant (5 days in the sponge group versus 6 days in the control group). Based on a refusal rate of 10 % in the intervention arm, a sample size of at least 82 patients per arm is needed to confirm this hypothesis. Because hospital stay generally does not have a Gaussian distribution, we decided to add 15 % in order to adjust the sample size for analysis with nonparametric tests [18]. Our final sample size was calculated to be 98 patients per arm.

### Statistical analysis

Data will be analyzed according to the intention-to-treat principle. Differences in continuous outcomes will be tested using independent samples t-tests or nonparametric equivalents, depending on the distribution. Chi squared tests (or Fishers Exact tests) will be used to test differences in categorical variables. Adjusted analyses will be performed in case of unbalanced randomization between the intervention and control arm. A *P* value of 0.05 is considered significant. Statistical analyses will be performed using Statistical Package for Social Sciences (SPSS) software (IBM SPSS Statistics for Windows, Armonk, NY: IBM Corp.).

### Safety reporting

Within the observational colorectal cancer cohort, serious adverse events (SAEs) are measured by an annual questionnaire. For the present study, serious adverse device events (SADEs) and study-specific SAEs are measured, including major complications, as an event within the first 30 days after sponge-assisted surgery resulting in any serious surgical complications or any medical event resulting in death or a life threatening situation. The sponsor will report these events within 15 days after notification. SADEs that result in death or are life threatening are reported within 7 days after the responsible investigator has first knowledge of it. SADEs and SAEs are reported on a Dutch web portal (www.toetsingonline.nl).

## Discussion

The SPONGE trial is the first randomized controlled trial to evaluate the impact of the use of the retractor sponge during laparoscopic colorectal surgery on duration of hospital stay and postoperative complications. Since no alternative for the Trendelenburg position has been available, the clinical relevance of the effects related to this position has never been investigated. Recently, results of a pilot study suggested that avoidance of the Trendelenburg position might reduce the postoperative hospital stay [10]. This effect could potentially be explained by a more stable perioperative hemodynamic status, which may make patients less prone to develop complications. Since this pilot study was a nonrandomized retrospective matched analysis, the risk of bias is considerable. Therefore, a randomized trial is warranted to estimate an unbiased effect of the Trendelenburg position on postoperative complications and hospital stay.

This trial is relevant because the number of (non-)surgical postoperative complications after colorectal surgery is substantial and result in a relatively high morbidity and mortality rate [19, 20]. Even though laparoscopic colorectal surgery is associated with certain benefits as a shorter hospital stay, less blood loss, and a faster recovery of bowel function, the risk on postoperative cardiopulmonary complications is not evidently lower than in open surgical procedure. A systematic review on nonsurgical complications following laparoscopic or open surgery for colorectal cancer showed no difference in the risk of pulmonary complications (pooled analysis for laparoscopy in colon cancer (OR 0.78, 95 % CI 0.53 to 1.13) and in rectal cancer (OR 1.19, 95 % CI 0.74 to 1.90)) [21]. Significantly fewer cardiac adverse events were observed in patients undergoing colectomy, but not in those undergoing rectal surgery (OR 0.28, 95 % CI 0.11 to 0.71 and OR 0.85, 95 % CI 0.41 to 1.71, respectively). This could be a result of the more extensive surgery, longer operation time, and higher risk on overall postoperative morbidity in rectal surgery [22]. Several studies presented the effects of the Trendelenburg position on perioperative cardiopulmonary parameters, but postoperative data are lacking [7, 8, 23–26]. Besides, the effect of a patient’s position as an independent factor is hard to evaluate because the perioperative hemodynamic parameters are partly influenced by pneumoperitoneum, which is the CO_2_ gas insufflation in the abdominal cavity [3].

The retractor sponge may provide two additional advantages. In the Trendelenburg position, cephalad excursion (sliding) of the patient while on the operation table can be problematic, particularly for obese patients or when the tables are positioned at steep angles. Sliding can cause skin and neuropathic injuries and can disturb the surgical procedure [27, 28]. Some devices used for sliding prevention, such as shoulder braces or headrests, can induce neuromuscular injuries, particularly brachial plexus injury, and are therefore not recommended [27]. In sponge-assisted surgery, patients are operated in horizontal position with no risk of sliding. Another potential benefit of a horizontal position is a more comfortable ergonomic position for the surgeon. The retractor sponge allows the surgeon to work with laparoscopic instruments in a horizontal line with the patient, which may reduce tension in the arms and neck. Unfortunately, we are not able to investigate these potential ergonomic benefits because this trial is performed at only one center with extensive experience in sponge-assisted surgery.

This study uses the cmRCT design. This design aims to overcome shortcomings of classic RCTs such as slow recruitment, disappointment bias in patients randomized to the control arm, and poor generalizability due to enrollment of only a small proportion of eligible patients [14, 29]. Trial recruitment within the PLCRC cohort is expected to be more efficient because patients are recruited from a large patient cohort, and only patients randomly selected for the intervention are informed about the intervention. In popular low risk interventions, such as the one used in the present study, the risk of disappointment bias or dropout in the control arm, due to a strong preference for the sponge intervention, is therefore diminished.

A challenging aspect of the cmRCT design is the fact that multiple trials are conducted simultaneously within one cohort. In patients eligible for multiple trials, interference of endpoints between trials might occur. As mentioned earlier, some of the patients eligible for the SPONGE trial will have participated in the RECTAL BOOST study [14]. Patients having received the boost intervention potentially have a higher risk on acute toxicity than patients who undergo standard chemoradiation [30], which could result in perioperative complications and thereby prolonged hospital stay. In order to balance the number of patients treated with the experimental boost intervention between both arms, stratified randomization is required. The main advantage of stratified randomization is the systematic balance of factors, which may influence outcome measures between treatment groups resulting in trial efficiency, increased power and less need for (biased) post-hoc analysis [31]. Furthermore, stratified randomization enables the possibility to detect potential interactions between the boost intervention and outcome measures of the SPONGE trial. On the other hand, the more trials conducted within one cohort, the more strata required for randomization in every new trial. This will result in complex trial coordination, treatment allocation, and trial administration. Having too much trial-induced strata may result in overstratification. This could lead to unfilled strata, unequal distribution of stratification factors, or the need for adjusted analysis [31–33]. In addition, it is yet unclear if there is such as a limit in number of interventions per patient within the cmRCT design. In our cohort, these new methodological challenges should be taken into account when a new trial is initiated. In the future, expertise of clinicians will be essential in the design of new cmRCT-based trials because overlap of endpoints must be considered in an early stage since this could affect potential recruitment rates and thereby the succeeding of a trial.

A limitation of this study is that the trial is performed in a single center by a surgeon experienced with sponges. In the near future, other centers may participate, which will enhance the generalizability of the results. In addition, the trial is performed unblinded. The pragmatic cmRCT design makes blinding impossible because information must be provided to randomly selected patients because they have to choose whether they want to undergo sponge-assisted or standard surgery. Clinicians cannot be blinded because perioperative use of the sponge must be reported in the operation report for safety reasons. To minimize observer bias in physicians responsible for discharge of the patients, we use an objective primary endpoint in the form of a discharge checklist. Both intervention arm and control patients will be assessed by this checklist provided to attending physicians. According to the cmRCT design, patients in the control arm are not aware of the fact that a sponge intervention is available, which may minimize disappointment bias.

In conclusion, this trial will evaluate the clinical effects of sponge-assisted laparoscopic colorectal surgery compared to the standard Trendelenburg positioning. Results of the trial will be applicable to other institutions performing laparoscopic colorectal surgery, as well as to other laparoscopic pelvic procedures that make use of the Trendelenburg position.

### Trial status

The trial started in November 2015 and is currently enrolling participants.

### Ethics approval

This study will be conducted according to the principles of the Declaration of Helsinki (64th WMA General Assembly, Fortaleza, Brazil, October 2013) and in accordance with the Medical Research Involving Human Subjects Act (WMO), the Good Clinical Practice guidelines, and this study protocol. The trial study protocol was approved by a Dutch institutional review board (“Medical research Ethics Committees United” (MEC-U)) on 5 December 2014 and is registered on clinicaltrials.gov under ClinicalTrials.gov NCT02574013.

### Open Access

This article is distributed under the terms of the Creative Commons Attribution 4.0 International License (http://creativecommons.org/licenses/by/4.0/), which permits unrestricted use, distribution, and reproduction in any medium, provided you give appropriate credit to the original author(s) and the source, provide a link to the Creative Commons license, and indicate if changes were made. The Creative Commons Public Domain Dedication waiver (http://creativecommons.org/publicdomain/zero/1.0/) applies to the data made available in this article, unless otherwise stated.

## Abbreviations

cmRCT: Cohort Multiple Randomized Controlled Trial
PLCRC: Prospective Data Collection Initiative on Colorectal Cancer (in Dutch: Prospectief Landelijk cohort ColoRectaal Carcinoom)
VAS: Visual Analogue Scale

## References

[1] Ferlay J, Soerjomataram I, Ervik M, Dikshit R, Eser S, Mathers C (2012). GLOBOCAN 2012 v1.0, Cancer Incidence and Mortality Worldwide: IARC CancerBase.

[2] Redactie. DICA jaarrapport, DSCA 2013 [Internet]. 2013. Available from: http://www.clinicalaudit.nl/jaarrapportage/2013/

[3] Gainsburg DM. Anesthetic concerns for robotic-assisted laparoscopic radical prostatectomy. Minerva Anestesiol. 2012;78(5):596-604. 22415437

[4] Schramm P, Treiber A-H, Berres M, Pestel G, Engelhard K, Werner C (2014). Time course of cerebrovascular autoregulation during extreme Trendelenburg position for robotic-assisted prostatic surgery. Anaesthesia.

[5] Mavrocordatos P, Bissonnette B, Ravussin P (2000). Effects of neck position and head elevation on intracranial pressure in anaesthetized neurosurgical patients: preliminary results. J Neurosurg Anesthesiol.

[6] Pinkney TD, King AJ, Walter C, Wilson TR, Maxwell-Armstrong C, Acheson AG (2012). Raised intraocular pressure [IOP] and perioperative visual loss in laparoscopic colorectal surgery: a catastrophe waiting to happen? A systematic review of evidence from other surgical specialities. Tech Coloproctol.

[7] Kadono Y, Yaegashi H, Machioka K, Ueno S, Miwa S, Maeda Y, et al. Cardiovascular and respiratory effects of the degree of head-down angle during robot-assisted laparoscopic radical prostatectomy. Int J Med Robot. 2013;9(1):17-22. 10.1002/rcs.1482 23348954

[8] Kalmar AF, Foubert L, Hendrickx JFA, Mottrie A, Absalom A, Mortier EP (2010). Influence of steep Trendelenburg position and CO[2] pneumoperitoneum on cardiovascular, cerebrovascular, and respiratory homeostasis during robotic prostatectomy. Br J Anaesth.

[9] Kilic OF, Börgers A, Köhne W, Musch M, Kröpfl D, Groeben H. Effects of steep Trendelenburg position for robotic-assisted prostatectomies on intra- and extrathoracic airways in patients with or without chronic obstructive pulmonary disease. Br J Anaesth. 2015;114(1):70-6. 10.1093/bja/aeu322 25236948

[10] J.S. Pawiroredjo, MD^1^N. Rijkers, BSc^1^A.B. Smits, MD P. The use of endoractor during laparoscopic colorectal surgery; a new solution? Pilot study. NVGE Voorjaarscongres [Internet]. NH Conference Centre Koningshof Veldhoven; 2014. Available from: https://www.nvge.nl/ewiseabstract2?func=dispatch&type=abstractsbeheer&action=viewAbstract&abstractId=MA6rMbTeF51b91_nI1igmA&congresId=YMUV2KCT4RZq1l64UElcQg

[11] Relton C, Torgerson D, Cathain AO, Nicholl J (2010). Rethinking pragmatic randomised controlled trials : introducing the “ cohort multiple randomised controlled trial ” design. BMJ.

[12] Young-Afat DA, Verkooijen HM, Van Gils CH, Van der Velden JM, Burbach J, Elias SG, et al. Staged-informed consent in the cohort multiple Randomized Controlled Trial design: Rethinking patient-centered informed consent to avoid pre-randomization. Epidemiology. 2016 Jan 6. [Epub ahead of print] 10.1097/EDE.0000000000000435 27035689

[13] Oken MM, Creech RH, Tormey DC, Horton J, Davis TE, McFadden ET (1982). Am J Clin Oncol.

[14] Burbach JPM, Verkooijen HM, Intven M, Kleijnen J-PJ, Bosman ME, Raaymakers BW (2015). RandomizEd controlled trial for pre-operAtive dose-escaLation BOOST in locally advanced rectal cancer [RECTAL BOOST study]: study protocol for a randomized controlled trial. Trials.

[15] Matsuoka S, Kikuchi I, Kitade M, Kumakiri J, Jinushi M, Tokita S (2011). Utility of an organ retraction sponge [endoractor] in gynecologic laparoscopic surgery. J Minim Invasive Gynecol.

[16] Delaney CP (2008). Outcome of Discharge Within 24 to 72 Hours After Laparoscopic Colorectal Surgery. Dis Colon Rectum.

[17] Iranmanesh P, Frossard JL, Mugnier-Konrad B, Morel P, Majno P, Nguyen-Tang T (2014). Initial Cholecystectomy vs Sequential Common Duct Endoscopic Assessment and Subsequent Cholecystectomy for Suspected Gallstone Migration. JAMA.

[18] Erich L. Lehmann. Nonparametrics : Statistical Methods Based on Ranks, Revised. ISBN = 978-0139977350 [Internet]. 1998. 76-81 p. Available from: http://www.graphpad.com/guides/prism/6/statistics/index.htm?stat_sample_size_for_nonparametric_.htm

[19] Hendren SK, Morris AM. Evaluating Patients Undergoing Colorectal Surgery to Estimate and Minimize Morbidity and Mortality. Surgical Clinics of North America [Internet]. Elsevier Inc; 2013 Feb [cited 2014 Apr 7]; 93[1]:1–20. Available from: http://linkinghub.elsevier.com/retrieve/pii/S0039610912001879 10.1016/j.suc.2012.09.005 23177062

[20] Böttger TC, Hermeneit S, Müller M, Terzic A, Rodehorst A, Elad L (2009). Modifiable surgical and anesthesiologic risk factors for the development of cardiac and pulmonary complications after laparoscopic colorectal surgery. Surg Endosc.

[21] Schiphorst AHW, Verweij NM, Pronk A, Borel Rinkes IHM, Hamaker ME. Non-surgical complications after laparoscopic and open surgery for colorectal cancer - A systematic review of randomised controlled trials. European journal of surgical oncology : the journal of the European Society of Surgical Oncology and the British Association of Surgical Oncology [Internet]. 2015 May 1 [cited 2015 Jun 8]; Available from: http://www.sciencedirect.com/science/article/pii/S0748798315003911 10.1016/j.ejso.2015.04.007 25980746

[22] Van der Pas MH, Haglind E, Cuesta MA, Fürst A, Lacy AM, Hop WC (2013). Laparoscopic versus open surgery for rectal cancer [COLOR II]: short-term outcomes of a randomised, phase 3 trial. Lancet Oncol.

[23] Danic MJ, Chow M, Alexander G, Bhandari A, Menon M, Brown M (2007). Anesthesia considerations for robotic-assisted laparoscopic prostatectomy: a review of 1,500 cases. J Robot Surg.

[24] Lestar M, Gunnarsson L, Lagerstrand L, Wiklund P, Odeberg-Wernerman S (2011). Hemodynamic perturbations during robot-assisted laparoscopic radical prostatectomy in 45° Trendelenburg position. Anesth Analg.

[25] Falabella A, Moore-Jeffries E, Sullivan MJ, Nelson R, Lew M (2007). Cardiac function during steep Trendelenburg position and CO2 pneumoperitoneum for robotic-assisted prostatectomy: a trans-oesophageal Doppler probe study. Int J Med Robot.

[26] Haas S, Haese A, Goetz AE, Kubitz JC (2011). Haemodynamics and cardiac function during robotic-assisted laparoscopic prostatectomy in steep Trendelenburg position. Int J Med Robot.

[27] Barnett JC, Hurd WW, Rogers RM, Williams NL, Shapiro SA (2007). Laparoscopic positioning and nerve injuries. J Minim Invasive Gynecol.

[28] Shveiky D, Aseff JN, Iglesia CB (2010). Brachial plexus injury after laparoscopic and robotic surgery. J Minim Invasive Gynecol.

[29] Mol L, Koopman M, van Gils CWM, Ottevanger PB, Punt CJA (2013). Comparison of treatment outcome in metastatic colorectal cancer patients included in a clinical trial versus daily practice in The Netherlands. Acta Oncol.

[30] Burbach JPM, den Harder AM, Intven M, van Vulpen M, Verkooijen HM, Reerink O (2014). Impact of radiotherapy boost on pathological complete response in patients with locally advanced rectal cancer: a systematic review and meta-analysis. Radiother Oncol.

[31] Kernan WN, Viscoli CM, Makuch RW, Brass LM, Horwitz RI (1999). Stratified randomization for clinical trials. J Clin Epidemiol.

[32] Simon R (1982). Patient subsets and variation in therapeutic efficacy. Br J Clin Pharmacol.

[33] Kahan BC, Morris TP (2012). Improper analysis of trials randomised using stratified blocks or minimisation. Stat Med.

